# Metformin regulates metabolic and nonmetabolic pathways in skeletal muscle and subcutaneous adipose tissues of older adults

**DOI:** 10.1111/acel.12723

**Published:** 2018-01-31

**Authors:** Ameya S Kulkarni, Erika F Brutsaert, Valentin Anghel, Kehao Zhang, Noah Bloomgarden, Michael Pollak, Jessica C Mar, Meredith Hawkins, Jill P Crandall, Nir Barzilai

**Affiliations:** ^1^ Division of Endocrinology Department of Medicine Albert Einstein College of Medicine Bronx NY USA; ^2^ Institute for Aging Research Albert Einstein College of Medicine Bronx NY USA; ^3^ Institute for Clinical and Translational Research Albert Einstein College of Medicine Bronx NY USA; ^4^ Department of Oncology McGill University Montreal QC Canada; ^5^ Department of Systems and Computational Biology Albert Einstein College of Medicine Bronx NY USA; ^6^ Australian Institute for Bioengineering and Nanotechnology University of Queensland Brisbane QLD Australia; ^7^ Diabetes Research Center Albert Einstein College of Medicine Bronx NY USA

**Keywords:** aging, biguanides, gene expression, metabolism, upstream regulators

## Abstract

Administration of metformin increases healthspan and lifespan in model systems, and evidence from clinical trials and observational studies suggests that metformin delays a variety of age‐related morbidities. Although metformin has been shown to modulate multiple biological pathways at the cellular level, these pleiotropic effects of metformin on the biology of human aging have not been studied. We studied ~70‐year‐old participants (*n* = 14) in a randomized, double‐blind, placebo‐controlled, crossover trial in which they were treated with 6 weeks each of metformin and placebo. Following each treatment period, skeletal muscle and subcutaneous adipose tissue biopsies were obtained, and a mixed‐meal challenge test was performed. As expected, metformin therapy lowered 2‐hour glucose, insulin AUC, and insulin secretion compared to placebo. Using FDR<0.05, 647 genes were differentially expressed in muscle and 146 genes were differentially expressed in adipose tissue. Both metabolic and nonmetabolic pathways were significantly influenced, including pyruvate metabolism and DNA repair in muscle and PPAR and SREBP signaling, mitochondrial fatty acid oxidation, and collagen trimerization in adipose. While each tissue had a signature reflecting its own function, we identified a cascade of predictive upstream transcriptional regulators, including mTORC1, MYC, TNF, TGFß1, and miRNA‐29b that may explain tissue‐specific transcriptomic changes in response to metformin treatment. This study provides the first evidence that, in older adults, metformin has metabolic and nonmetabolic effects linked to aging. These data can inform the development of biomarkers for the effects of metformin, and potentially other drugs, on key aging pathways.

## INTRODUCTION

Emerging interest in interventions that target the molecular processes of aging has resulted in the testing of numerous agents that slow aging and increase healthspan in model systems (Kennedy et al., 2014). A major barrier in translating these findings from model organisms to humans is the long human lifespan and lack of consensus on appropriate surrogate markers for improvements in human aging.

Metformin, a biguanide derived from *Galega officinalis*, has been used to treat diabetes for over 60 years. It is the first line treatment for type 2 diabetes because of its low cost, safety profile and effectiveness in decreasing hepatic gluconeogenesis and improving insulin sensitivity. It also modulates cellular processes associated with aging including inflammation, oxidative stress, cellular senescence, autophagy, and apoptosis (Barzilai, Crandall, Kritchevsk, & Espeland, 2016). However, it is not clear whether metformin targets these processes individually, or they are a result of metformin “fixing” cellular aging in a more fundamental way. Some of the primary effects are thought to be mediated by metformin's actions on nutrient sensing and utilization, which include inhibition of mitochondrial complex I and activation of AMP kinase, although less well‐characterized mechanisms are likely to be involved (Rena, Hardie, & Pearson, 2017). In addition to promising molecular changes induced by metformin, there is evidence that it improves rodent lifespan and healthspan indices and induces transcriptomic changes mimicking those of caloric restriction (Martin‐Montalvo et al., 2013). Importantly, human studies suggest that metformin may reduce the incidence of age‐related diseases, including cardiovascular disease and cancer and decrease mortality, independently of its effects on glucose metabolism (Aroda et al., 2017; Bannister et al., 2014; Campbell, Bellman, Stephenson, & Lisy, 2017; Griffin, Leaver, & Irving, 2017).

In this crossover study design, each subject serves as their own placebo‐treated control, increasing our power to detect metformin‐associated changes in a small sample of adults and allowing us to address the following questions: (i) Does exposure to 6 weeks of metformin induce relevant physiological and transcriptomic changes in human muscle and adipose tissues? (ii) Are these changes tissue‐specific? (iii) What biological pathways are affected by metformin action? (iv) Are there common upstream regulators that are responsible for these transcriptomic changes?

Baseline characteristics of the subjects who completed the study are shown in Table S1, and randomization groups were well‐matched. Plasma metformin concentration was 18 μM ± 6 at 165 mins after metformin ingestion**.** Metformin therapy decreased 2‐hour glucose over placebo, insulin AUC, and insulin secretion using C‐peptide deconvolution and improved low‐density lipoprotein and triglycerides (Table 1). These findings indicate that metformin can induce favorable metabolic changes in older adults with impaired glucose tolerance, the most common form of glucose dysregulation in this age group.

**Table 1:** Cardio‐metabolic variables at the end of the metformin and placebo treatment periods. The *p*‐values in bold and highlighted with a * are significant at a threshold of .05

	Metformin	Placebo	*p*‐value
Weight (kg)	82.9 ± 15	84.2 ± 15	.012*
Systolic BP (mmHg)	139 ± 17	139 ± 11	.9
Diastolic BP (mmHg)	75 ± 9.7	77 ± 12	.35
Fasting glucose (mg/dl)	103 ± 8.3	106 ± 8.5	.36
Peak postmeal glucose (mg/dl)	159 ± 46	177 ± 30	.14
2 hrs glucose (mg/dl)	145 ± 37	166 ± 31	.04*
Glucose AUC _0‐180_ ((mg/dl) * min)	24350 ± 5223	26311 ± 3920	.27
Insulin AUC _0‐180_ ((μu/ml) * min)	7605 ± 3742	10995 ± 6098	.005*
HOMA‐IR	2.6 ± 1.3	2.9 ± 1.5	.27
Matsuda 0–120	5.6 ± 3.9	4.1 ± 2.1	.11
Insulin secretion (μu/ml)	13.8 ± 6.2	17.7 ± 6.2	.004*
HbA1c (%)	5.7 ± 0.4	5.8 ± 0.3	.22
hs‐CRP (mg/l)	1.8 ± 1.3	2.0 ± 1.7	.26
Adiponectin (μg/ml)	10 ± 6.7	12 ± 6.5	.14
IGF‐1 (ng/ml)	127 ± 42	133 ± 43	.23
Total cholesterol (mg/dl)	153 ± 33	165 ± 32	.06
HDL (mg/dl)	46 ± 13	47 ± 14	.62
LDL (mg/dl)	86 ± 22	102 ± 40	.03*
Triglycerides (mg/dl)	83 ± 23	101 ± 50	.03*
MAP (mmHg)	97 ± 11	98 ± 10	.52
RHI (fasting)	1.9 ± 0.4	2 ± 0.4	.29
Augmentation index	25 ± 12	28 ± 16	.4

**Figure 1:**
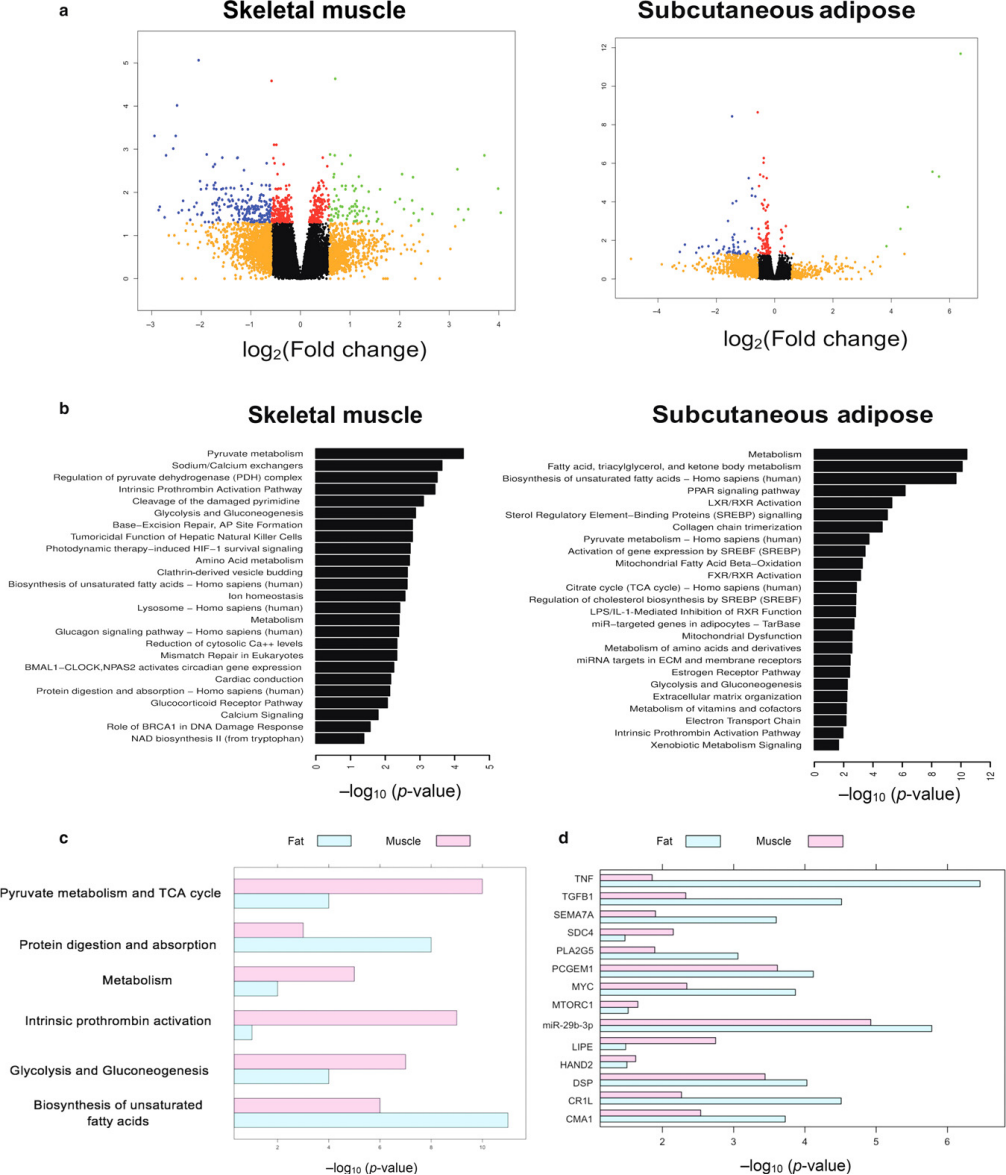
Metformin influences tissue‐specific metabolic and nonmetabolic genes and pathways in elderly humans: (a) volcano plots for 647 differentially expressed genes (DEG) in muscle and 146 DEG in adipose, with metformin treatment. (b) Pathways overrepresented in muscle and adipose‐specific DEG using ConsensusPathDB and ingenuity pathway analysis; (c) overrepresented pathways common to muscle and adipose; (d) predictive upstream regulators common to muscle and adipose, using IPA

Differential gene expression analysis between metformin and placebo treatments revealed 647 genes in muscle and 146 genes in adipose tissues (FDR < 0.05) (Figure 1a, Data S5, S6). Of these, 15 genes were common to both tissues, with 11 genes showing consistent up/downregulation in both muscle and adipose, while four genes were upregulated in muscle but downregulated in adipose (Table S2).

To examine whether these transcriptomic changes were relevant to aging, we intersected the differentially expressed genes with a list of aging genes from GenAge database (Tacutu et al., 2013). Four muscle‐specific genes *PCK1*,* E2F1*,* PARP1,* and *RET* and two adipose‐specific genes *SDHC* and *CETP* intersected with GenAge list. In addition, we found an enrichment of mitochondrial genes and genes coding for nuclear encoded mitochondrial proteins from MitoCarta 2.0, within the differentially expressed genes specific to adipose tissue (*p* = 6.02e−10, by one‐sided Fisher's exact test).

In muscle, pyruvate metabolism, DNA base excision and mismatch repair, NAD biosynthesis, and BRCA role in DNA damage response were some of the key pathways significantly overrepresented (Figure 1b, Table S3). Consistent with its regulation by caloric restriction, *PCK1* upregulation by metformin may mediate improvements in healthspan indices, as seen in mice (Hakimi et al., 2007). *PARP1* downregulation raises an intriguing possibility that metformin's effect on mitochondrial pathways may be mediated in part through the NAD^+^‐SIRT1‐PGC1α axis or via AMPK activation (Shang et al., 2016). The downregulation of proto‐oncogene *RET* and transcription factor *E2F1* supports metformin's previously identified antitumoral role in arresting cell cycle (Alimova et al., 2009). Pathway analysis provided evidence that metformin affects pyruvate metabolism, leading to muscle energy homeostasis and metabolic flexibility. Moreover, pyruvate dehydrogenase complex, linking fatty acid metabolism, glucose metabolism, and TCA cycle, has been suggested as a therapeutic target for age‐related diseases (Stacpoole, 2012). Our novel findings also indicate that metformin regulates MutS family genes *MSH2* and *MSH3* involved in DNA mismatch repair, a postreplicative correction pathway that declines with age, demonstrating the profound effects metformin on several biological aging pathways.

In adipose, metformin primarily affects fatty acid metabolism, biosynthesis of unsaturated fatty acid, PPAR and SREBP signaling, LXR/RXR activation, collagen chain trimerization, and ECM remodeling (Figure 1b, Table S4). Metformin is known to alter fatty acid metabolism similar to other interventions that extend lifespan in model organisms (Barzilai, Huffman, Muzumdar, & Bartke, 2012). Age‐related deposition of ECM and fibrosis contributes to metabolic dysregulation of adipose tissue (Sun, Kusminski, & Scherer, 2011). Our finding that metformin decreased expression of multiple collagen genes suggests that metformin may attenuate ECM and thus improve aging‐associated dysregulation of adipose tissue (Luo et al., 2016).

Although there were only 15 differentially expressed genes common to both tissues, pathway analysis uncovered six pathways overrepresented in both tissues in response to metformin (Figure 1c). Several tissue‐specific upstream regulators involved in both metabolic and nonmetabolic pathways were predicted to be contributing to metformin‐associated transcriptomic changes (Data S7/S8). In muscle, miRNAs and transcriptional regulators were predicted to be activated, while inflammatory mediators were predicted to be inhibited. In adipose, ligand‐dependent nuclear receptors were predicted to be inhibited and *SMAD7* and *miR29b‐3P* were predicted to be activated. We identified 17 common predictive upstream regulators of the differentially expressed genes between muscle and adipose (Figure 1d), including *miR29b‐3p* and *CR1L* predicted to be activated, while *PCGEM1, TGFß1,* and *TNF* predicted to be inhibited while *mTORC1* predicted to be inhibited only in the muscle. The upstream regulators influenced by metformin may mediate the multitude of downstream gene expression changes. Interestingly, the common upstream regulators of the differentially expressed genes in both muscle and adipose include inflammatory mediators, supporting the recently discussed role of metformin in improving the adverse milieu associated with “inflammaging” (Cameron et al., 2016). miR29b, identified as a common upstream regulator in both tissues, is associated with age‐related diseases and appears to play a role in DNA damage response (Ugalde et al., 2011). Other regulators include *MYC*, an oncogene, inversely regulating mammalian lifespan and healthspan and *mTORC1*, a key regulator of aging and autophagy (Hofmann et al., 2015; Johnson, Rabinovitch, & Kaeberlein, 2013).

In summary, this study showed that 6 weeks of metformin can improve age‐associated metabolic derangements in glucose intolerant older adults. While metformin had tissue‐specific effects on gene expression in muscle and adipose, it is predicted to target common upstream transcriptional regulators related to pathways implicated in aging. In fact, metformin influenced not only metabolic genes and pathways, but also collagen and mitochondrial genes in adipose, and DNA repair genes in muscle, which emphasize its targeting of multiple hallmarks of aging. However, the gene expression changes may not fully explain metformin's action on enzymatic activity, and further mechanistic studies are essential to substantiate metformin's role in targeting aging. These findings remain to be validated in other tissues and study designs and do not yet allow us to identify the primary site of action of metformin, which then may trigger the observed changes in gene expression. If indeed, aging can be reversed at cellular and organ levels, development of relevant biomarkers (e.g., gene expression) will advance the identification of other, perhaps more potent drugs to target aging.

## CONFLICT OF INTEREST

None to declared.
