# Supplementary material for: Metformin regulates metabolic and nonmetabolic pathways in skeletal muscle and subcutaneous adipose tissues of older adults
Source: Aging Cell. 2018 Jan 31;17(2):e12723. doi: 10.1111/acel.12723 (PMC5847877; doi:10.1111/acel.12723)
Supplement: Supplementary file 1 [file ACEL-17-e12723-s001.docx]

**Supplemental Information**

**Experimental Procedures**

**Subjects**
The study was approved by the Albert Einstein College of Medicine Institutional Review Board and written informed consent was obtained from all subjects. Subjects were men and women aged 60 and older, with impaired glucose tolerance (IGT) based on 75g OGTT (fasting plasma glucose < 126 mg/dl, 2-hr glucose between 140 – 199 mg/dl), without a known history of diabetes or prior or current treatment with anti-hyperglycemic agents. Exclusions included serious chronic or acute illness, including active or recent cancer, (other than non-melanoma skin cancer), symptomatic heart failure, chronic obstructive pulmonary disease, inflammatory conditions, significant liver disease or renal disease, recent (within 3 months) cardiovascular event (MI, revascularization or stroke); prior bariatric surgery or cigarette smoking. Also exclusionary was use of drugs known to influence glucose metabolism (e.g., systemic glucocorticoids), antioxidant vitamins, warfarin or antiplatelet drugs (other than aspirin). Forty-four individuals over 60 years old were screened with an OGTT, 17 were eligible by OGTT criteria of impaired glucose tolerance (IGT), and 16 individuals consented to randomization. 2 participants withdrew consent after study initiation due to inability to complete the biopsy procedure, and 14 completed the study.

**Study design**

The study was a randomized, double-blind, placebo controlled, crossover study. Metformin was introduced at 500 mg twice daily, to be taken with morning and evening meal, and increased incrementally to 2000 mg daily at the end of 2 weeks to minimize gastrointestinal side effects. Participants with adverse effects from metformin were permitted to participate in the study on a lower dose of metformin. A placebo capsule containing lactose was made by the Montefiore Medical Center research pharmacy. Randomization, blinding, and dispensing was also conducted by the research pharmacy of Montefiore Medical Center. Subjects were instructed to maintain their usual dietary and physical activity patterns. The compliance rate, defined as the proportion of tablets ingested relative to the intended number, was calculated based on remaining tablets returned at the end of the treatment period.

**Study visits and interventions**

Following a screening visit, the study consisted of two randomly assigned 6-week

treatment periods (metformin and placebo). Following a 2-week washout period, the participants crossed-over to the other intervention for the second 6-week treatment period.

**Standard Mixed-Meal Test**

At the end of each 6-week treatment period, a standard mixed meal test was conducted. Subjects were instructed to fast overnight and were given a standard breakfast consisting of 110 g carbohydrates, 20 g protein and 20 g fat. Blood sampling for glucose and insulin levels was performed fasting (time 0) and 30, 60, 90, 120, 150 and 180 minutes following the mixed-meal through an indwelling catheter. The assigned treatment (metformin or placebo) was administered with the meal. Insulin sensitivity was estimated using homeostatic model assessment (HOMA-IR) and Matsuda index. β-cell function was assessed with the oral disposition index (DI_O_) calculated using the formula: (∆I_0- 30_/∆_G0-30_) * (1/I_0_). Insulin and glucose area under the curve (AUC) were calculated using the trapezoidal method.

Glucose, hemoglobin A1c (HbA1c), lipoproteins, insulin (radioimmunoassay), C-peptide (radioimmunoassay), high sensitivity C-reactive protein (latex-enhanced turbidimetric assay), IGF-1 (radioimmunoassay) and adiponectin (radioimmunoassay; Linco) assays were performed at the core laboratories of the Einstein Institute for Clinical and Translational Research. Blood chemistries, complete blood count and urinalysis were performed in the clinical laboratories of Montefiore Medical Center. Levels of metformin were measured in the plasma of 7 subjects at 0, 30, 60, 120 and 180 minutes during the standard mixed-meal test in the metformin treatment period. The concentrations of metformin levels in plasma were determined for the 7 subjects using a previously described protocol, in the laboratory of Dr. Michael Pollak, at McGill University, Montreal, Canada. (Chandel et al. 2016).

Endothelial Function Testing was performed fasting, using reactive hyperemia peripheral arterial tonometry (RH-PAT), which measures arterial pulse wave amplitude in the finger before and after 5 minutes of blood flow occlusion using a standard blood pressure cuff (EndoPAT; Itamar Medical). The reactive hyperemia index (RHI) is the ratio of the average pulse amplitude in the post-hyperemic phase divided by the average baseline amplitude, with normalization to the signal in the control arm to compensate for any systemic changes. Augmentation index (a measure of arterial stiffness) was also derived from the PAT signal.

**Skeletal muscle and subcutaneous adipose samples**

Skeletal muscle and subcutaneous adipose biopsies were performed in the fasting state. A muscle sample of ~50-100 mg was obtained with a spring-loaded biopsy needle (Bard Instruments) in the mid-thigh region (*vastus lateralis*) following local anesthesia extending into the muscle area. Subcutaneous adipose sample of ~800-1000 mg was obtained from the abdominal periumbilical region following a local anesthesia. The samples were immediately homogenized in Trizol, frozen in liquid nitrogen and stored at -80°C for subsequent mRNA extraction and analysis of gene expression.

**RNA-Seq Analysis**

**Library preparation, preprocessing and alignment**

For each muscle and adipose sample, total RNA was extracted using QIAGEN’s RNeasy Mini kit. Samples showing minimal degradation, as measured by RNA Integrity Number (RIN > 7) were processed for library preparation and sequenced at the Genomics Core of the Albert Einstein College of Medicine, using multiplexed 100bp single-end sequencing on Illumina HiSeq2500 on the Illumina HiSeq 2500 (<http://www.illumina.com/technology/mrna_seq.ilmn)> using a previously described paired-end 101 BP dual indexing protocol (Pollack et al. 2017).

Raw sequence reads were preprocessed using WASP 3.0, an in-house pipeline and FastQC was used for quality control (Andrews 2010, McLellan et al. 2012). The raw FASTQ files were trimmed for adapter sequences using Trim Galore! RSEM (RNA Sequencing by Expectation maximization) algorithm (version 1.2.25) in conjunction with STAR aligner (version 2.4.2a) were used to map the raw reads to GRCh38 build of the reference human genome with transcript annotations downloaded from GENCODE (Li and Dewey 2011, Harrow et al. 2012). The RNA-Sequencing data for muscle and adipose samples in both placebo and metformin conditions are accessible in the Gene Expression Omnibus (GEO) Database under accession code GSE107894.

**Statistical Analysis**

Data for cardio-metabolic variables are presented as mean ±SD for baseline values. Placebo vs. metformin variables (e.g. peak and AUC glucose, insulin, Matsuda index, RHI, etc.) were compared using a paired t-test. If the data were not normally distributed based on a visual inspection of the histogram and qq-plot, non-parametric test (Wilcoxon’s test) was used. The possibility of a carry-over effect between treatment periods was analyzed using a mixed effects model. Data analysis was performed using STATA version 14.

All statistical analyses for gene expression data were carried out using the R statistical software (version 3.4.0). Genes with a count-per-million value of <1 in two or more libraries were filtered out for low expression. Differential gene expression analysis between the placebo and metformin treated cohorts, was performed using the edgeR package (version 3.18.1) (McCarthy et al. 2012). Due to the nature of the crossover experimental design, a genewise negative binomial generalized linear model was fit to the read counts of each gene, using covariates for *treatments, subject ID* and *visit number* for the corresponding treatment, as blocking variables. Differentially expressed genes were identified using a likelihood ratio test for the *treatments* coefficient. This allowed for comparing effects of metformin versus placebo, using experimental subjects and visit times, that are as similar as possible, so that the treatment differences on gene expression stand out clearly. P-values were adjusted for multiple hypotheses testing using the Benjamini-Hochberg method. The FDR-adjusted p-value of < 0.05 was used as the statistical significance threshold for identifying differentially expressed genes. The enrichment for mitochondrial genes and nuclear encoded mitochondrial genes obtained from MitoCarta 2.0 (Calvo et al. 2016) as well as aging-relevant genes from GenAge (Tacutu et al. 2013), was calculated using one-sided Fisher’s exact test, in R.

**Pathway and upstream regulator Analysis**

All differentially expressed genes (FDR < 0.05) were imported to ConsensusPathDB (CBDB) and Ingenuity Pathway Analysis (version 01-12), to carry out gene set overrepresentation analysis and to identify common upstream regulators. Pathways defined by KEGG, REACTOME, Humancyc, Wikipathways and PharmGKB were selected in ConsensusPathDB to analyze overrepresented gene sets.

**Supplemental Table S1:** Baseline characteristics of study participants. Total, and separated by randomization group.

Randomization group 1 received metformin in the first treatment period and placebo in the second treatment period. Randomization group 2 received treatments in opposite order. All continuous data expressed in mean ± standard deviation. All count data expressed in number (% frequency)

**Supplemental Table S2:** Fifteen differentially expressed genes common between muscle and adipose after metformin treatment and their tissue-specific log_2_(Fold Change) and false discovery rate adjusted p-value.

11 genes showed consistent fold change, whereas 4 genes *RTN3, NSMCE4A, GNS* and *HACD2* were upregulated in muscle and downregulated in adipose. Gene names from HUGO gene symbols were obtained using HGNC multi-symbol checker.

**Supplemental Table S3:** Pathways overrepresented in metformin induced DEGs in muscle, annotation set size, overlapping number of genes (%), p-value of overlap and pathway annotation sources from ConsensuspathDB or Ingenuity Pathway Analysis (IPA).

**Supplemental Table S4:** Pathways overrepresented in metformin induced DEGs in adipose, annotation set size, overlapping number of genes (%), p-value of overlap and pathway annotation sources from ConsensuspathDB or Ingenuity Pathway Analysis (IPA)

**Supplemental Data S5, S6:** Metformin-induced differentially expressed genes in the muscle (S5) and adipose (S6), along with log_2_(Fold Change) and False-discovery-rate adjusted p-value.

All genes with FDR<0.05 are defined as differentially expressed. The genes are sorted according to the log_2_(FC) value and gene names are obtained from HGNC multi-symbol checker.

**Supplemental Data S7, S8:** Ingenuity Pathway Analysis predicted upstream regulators of differentially expressed genes in muscle (S7) and adipose (S8), p-value of overlap and predicted Activation z-score.

A positive activation z-score indicates that the regulator is activated and a negative z-score indicates that the regulator is inhibited.

**References**

Andrews, S. (2010). FastQC: a quality control tool for high throughput sequence data

Calvo, S. E., K. R. Clauser and V. K. Mootha (2016). "MitoCarta2.0: an updated inventory of mammalian mitochondrial proteins." Nucleic Acids Res **44**(D1): D1251-1257

Chandel, N. S., D. Avizonis, C. R. Reczek, S. E. Weinberg, S. Menz, R. Neuhaus, S. Christian, A. Haegebarth, C. Algire and M. Pollak (2016). "Are Metformin Doses Used in Murine Cancer Models Clinically Relevant?" Cell Metab **23**(4): 569-570

Harrow, J., A. Frankish, J. M. Gonzalez, E. Tapanari, M. Diekhans, F. Kokocinski, B. L. Aken, D. Barrell, A. Zadissa, S. Searle, I. Barnes, A. Bignell, V. Boychenko, T. Hunt, M. Kay, G. Mukherjee, J. Rajan, G. Despacio-Reyes, G. Saunders, C. Steward, R. Harte, M. Lin, C. Howald, A. Tanzer, T. Derrien, J. Chrast, N. Walters, S. Balasubramanian, B. Pei, M. Tress, J. M. Rodriguez, I. Ezkurdia, J. van Baren, M. Brent, D. Haussler, M. Kellis, A. Valencia, A. Reymond, M. Gerstein, R. Guigó and T. J. Hubbard (2012). "GENCODE: The reference human genome annotation for The ENCODE Project." Genome Research **22**(9): 1760-1774.

Li, B. and C. N. Dewey (2011). "RSEM: accurate transcript quantification from RNA-Seq data with or without a reference genome." BMC Bioinformatics **12**: 323.

McCarthy, D. J., Y. Chen and G. K. Smyth (2012). "Differential expression analysis of multifactor RNA-Seq experiments with respect to biological variation." Nucleic Acids Res **40**(10): 4288-4297.

McLellan, A. S., R. A. Dubin, Q. Jing, P. O. Broin, D. Moskowitz, M. Suzuki, R. B. Calder, J. Hargitai, A. Golden and J. M. Greally (2012). "The Wasp System: an open source environment for managing and analyzing genomic data." Genomics **100**(6): 345-351

Pollack, R. M., N. Barzilai, V. Anghel, A. S. Kulkarni, A. Golden, P. O'Broin, D. A. Sinclair, M. S. Bonkowski, A. J. Coleville, D. Powell, S. Kim, R. Moaddel, D. Stein, K. Zhang, M. Hawkins and J. P. Crandall (2017). "Resveratrol Improves Vascular Function and Mitochondrial Number but Not Glucose Metabolism in Older Adults." J Gerontol A Biol Sci Med Sci.

**Supplemental Table S1**

|  | **Both groups, n=14** | **Randomization group 1, n=7** | **Randomization group 2, n=7** | **P- value** |
| --- | --- | --- | --- | --- |
| Age | 71 ± 6.4 | 69 ± 6.7 | 74 ± 5.4 | 0.15 |
| Race black (%) | 4(29) | 3 (42) | 1 (14) | 0.24 |
| Gender female (%) | 6 (43%) | 3 (43) | 3 (43) | 1.0 |
| Weight in kg | 85 ± 16 | 83 ±16 | 86± 17 | 0.78 |
| Waist circumference | 98 ± 8.7 | 95 ±10 | 101 ± 6.3 | 0.21 |
| BMI | 29 ± 3.6 | 28 ±3.3 | 31 ± 3.6 | 0.14 |
| Fasting glucose | 103 ± 11 | 105 ± 12 | 101± 12 | 0.61 |
| 2-hour glucose | 162 ± 22 | 155 ±14 | 169 ±27 | 0.25 |
| eGFR | 74 ± 13.9 | 79 ±13 | 69± 13 | 0.17 |
| Antihypertensive treatment, n (%) | 9 (64) | 4(57) | 5(71) | 0.58 |
| Statin therapy, n (%) | 4 (29) | 2(29) | 2(29) | 1.0 |
| Aspirin therapy, n (%) | 4 (29) | 2(29) | 2(29) | 1.0 |

**Supplemental Table S2**

| **Gene symbol** | **Gene name** | **Muscle** | | **Adipose** | |
| --- | --- | --- | --- | --- | --- |
|  |  | **log_2_(FC)** | **FDR** | **log_2_(FC)** | **FDR** |
| *BMP6* | bone morphogenetic protein 6 | 0.45 | 0.02 | 0.39 | 0.002 |
| *GALNT15* | polypeptide N-acetylgalactosaminyltransferase 15 | 0.36 | 7.79E-05 | 0.26 | 0.004 |
| *HIVEP1* | human immunodeficiency virus type I enhancer binding protein 1 | 0.33 | 4.14E-05 | 0.25 | 0.007 |
| *RPL36AL* | ribosomal protein L36a like | 0.26 | 0.001 | 0.21 | 0.008 |
| *RTN3* | reticulon 3 | 0.25 | 0.0008 | -0.23 | 0.02 |
| *NSMCE4A* | NSE4 homolog A, SMC5-SMC6 complex component | 0.34 | 0.0005 | -0.24 | 0.04 |
| *GNS* | glucosamine (N-acetyl)-6-sulfatase | 0.22 | 0.0005 | -0.26 | 0.0002 |
| *COL15A1* | collagen type XV alpha 1 chain | -0.33 | 0.0003 | -0.30 | 0.049 |
| *HACD2* | 3-hydroxyacyl-CoA dehydratase 2 | 0.28 | 0.001 | -0.31 | 0.02 |
| *COL1A2* | collagen type I alpha 2 chain | -0.40 | 0.0002 | -0.35 | 0.02 |
| *NREP* | neuronal regeneration related protein | -0.35 | 9.39E-05 | -0.38 | 0.0098 |
| *COL3A1* | collagen type III alpha 1 chain | -0.46 | 5.87E-06 | -0.49 | 3.88E-06 |
| *MXRA5* | matrix remodeling associated 5 | -0.39 | 0.0006 | -0.58 | 2.27E-09 |
| *SLC2A5* | solute carrier family 2 member 5 | -0.52 | 0.0001 | -1.32 | 9.12E-05 |
| *CTD-2201\|18.1* | Uncharacterized | -1.29 | 0.0002 | -0.56 | 0.02 |

**Supplemental Table S3**

| **Pathway overrepresentation in muscle** | | | | |
| --- | --- | --- | --- | --- |
|  |  |  |  |  |
| **Pathway** | **Set size** | **Overlap** | **p-value** | **Source** |
| Pyruvate metabolism | 28 | 6 (21.4%) | 5.67E-05 | Reactome |
| Sodium/Calcium exchangers | 13 | 4 (30.8%) | 0.000234 | Reactome |
| Regulation of pyruvate dehydrogenase (PDH) complex | 14 | 4 (28.6%) | 0.000321 | Reactome |
| Intrinsic Prothrombin Activation pathway | 29 | 5 (17.2%) | 0.000371 | IPA |
| Glycolysis and Gluconeogenesis | 49 | 6 (12.2%) | 0.00135 | Wikipathways |
| Base-Excision Repair, AP Site Formation | 10 | 3 (30%) | 0.00165 | Wikipathways |
| Tumoricidal function of hepatic Natural Killer cells | 24 | 4 (16.7%) | 0.00167 | IPA |
| Photodynamic therapy-induced HIF-1 survival signaling | 36 | 5 (13.9%) | 0.00193 | Wikipathways |
| Amino Acid metabolism | 91 | 8 (8.8%) | 0.00203 | Wikipathways |
| Clathrin derived vesicle budding | 73 | 7 (9.6%) | 0.00233 | Reactome |
| Biosynthesis of unsaturated fatty acids - Homo sapiens (human) | 23 | 4 (17.4%) | 0.00237 | KEGG |
| Ion homeostasis | 56 | 6 (10.7%) | 0.00271 | Reactome |
| Lysosome - Homo sapiens (human) | 123 | 9 (7.3%) | 0.00383 | KEGG |
| Metabolism | 2035 | 69 (3.4%) | 0.00399 | Reactome |
| Glucagon signaling pathway - Homo sapiens (human) | 103 | 8 (7.8%) | 0.00413 | KEGG |
| Mismatch repair in eukaryotes | 16 | 3 (18.8%) | 0.00466 | IPA |
| Reduction of cytosolic Ca++ levels | 14 | 3 (21.4%) | 0.00466 | Reactome |
| Signaling by Retinoic Acid | 44 | 5 (11.4%) | 0.00472 | Reactome |
| BMAL1-CLOCK, NPAS2 activates circadian gene expression | 15 | 3 (20%) | 0.00571 | Wikipathways |
| Protein digestion and absorption - Homo sapiens (human) | 90 | 7 (7.8%) | 0.00746 | KEGG |
| Glucocorticoid Receptor Pathway | 71 | 6 (8.5%) | 0.00875 | Wikipathways |
| Calcium Signaling Pathway | 178 | 9 (5.1%) | 0.016 | IPA |
| Role of BRCA in DNA Damage Response | 78 | 5 (6.4%) | 0.0277 | IPA |
| NAD Biosynthesis from Tryptophan | 15 | 2 (13.3%) | 0.0413 | IPA |

**Supplemental Table S4**

| **Pathway overrepresentation in adipose tissue** | | | | |
| --- | --- | --- | --- | --- |
|  |  |  |  |  |
| **Pathway** | **Set size** | **Overlap** | **p-value** | **Source** |
| Lysine degradation - Homo sapiens (human) | 59 | 3 (5.1%) | 0.009556243 | KEGG |
| Electron Transport Chain | 103 | 4 (3.9%) | 0.00721071 | Wikipathways |
| Metabolism of vitamins and cofactors | 164 | 5 (3.1%) | 0.006927664 | Reactome |
| AGE-RAGE signaling pathway in diabetic complications - Homo sapiens (human) | 101 | 4 (4%) | 0.006733903 | KEGG |
| Extracellular matrix organization | 295 | 7 (2.4%) | 0.006222549 | Reactome |
| Platelet Aggregation Inhibitor Pathway, Pharmacodynamics | 49 | 3 (6.1%) | 0.00571199 | PharmGKB |
| Glycolysis and Gluconeogenesis | 49 | 3 (6.1%) | 0.00571199 | Wikipathways |
| Hematopoietic Stem Cell Differentiation | 47 | 3 (6.4%) | 0.005081052 | Wikipathways |
| linolenate biosynthesis | 14 | 2 (14.3%) | 0.004687225 | HumanCyc |
| Vitamin B5 (pantothenate) metabolism | 14 | 2 (14.3%) | 0.004687225 | Reactome |
| Estrogen Receptor Pathway | 13 | 2 (15.4%) | 0.004037072 | Wikipathways |
| Binding and Uptake of Ligands by Scavenger Receptors | 42 | 3 (7.1%) | 0.003694662 | Reactome |
| miRNA targets in ECM and membrane receptors | 42 | 3 (7.1%) | 0.003694662 | Wikipathways |
| Tryptophan metabolism - Homo sapiens (human) | 40 | 3 (7.5%) | 0.003213908 | KEGG |
| Metabolism of amino acids and derivatives | 328 | 8 (2.5%) | 0.002845814 | Reactome |
| miR-targeted genes in adipocytes - TarBase | 34 | 3 (8.8%) | 0.002010098 | Wikipathways |
| Regulation of cholesterol biosynthesis by SREBP (SREBF) | 31 | 3 (9.7%) | 0.001534405 | Reactome |
| Citrate cycle (TCA cycle) - Homo sapiens (human) | 30 | 3 (10%) | 0.001393368 | KEGG |
| Glycerolipid metabolism - Homo sapiens (human) | 59 | 4 (6.8%) | 0.000950736 | KEGG |
| Triacylglyceride Synthesis | 24 | 3 (12.5%) | 0.000717371 | Wikipathways |
| Mitochondrial Fatty Acid Beta-Oxidation | 22 | 3 (13.6%) | 0.000551727 | Reactome |
| Protein digestion and absorption - Homo sapiens (human) | 90 | 5 (5.6%) | 0.000542731 | KEGG |
| Valine, leucine and isoleucine degradation - Homo sapiens (human) | 48 | 4 (8.3%) | 0.000432563 | KEGG |
| Tryptophan metabolism | 46 | 4 (8.7%) | 0.000366903 | Wikipathways |
| Activation of gene expression by SREBF (SREBP) | 19 | 3 (15.8%) | 0.000352808 | Wikipathways |
| Composition of Lipid Particles | 4 | 2 (50%) | 0.000324379 | Wikipathways |
| Fatty acid degradation - Homo sapiens (human) | 44 | 4 (9.1%) | 0.000308699 | KEGG |
| Alzheimer’s disease - Homo sapiens (human) | 171 | 4 (4.1%) | 0.000277427 | KEGG |
| Pyruvate metabolism - Homo sapiens (human) | 39 | 4 (10.3%) | 0.000192417 | KEGG |
| Fatty Acid Beta Oxidation | 34 | 4 (11.8%) | 0.000111614 | Wikipathways |
| Collagen chain trimerization | 47 | 5 (10.6%) | 2.43E-05 | Reactome |
| Sterol Regulatory Element-Binding Proteins (SREBP) signaling | 68 | 6 (8.8%) | 1.08E-05 | Wikipathways |
| PPAR signaling pathway - Homo sapiens (human) | 72 | 7 (9.7%) | 9.89E-07 | KEGG |
| Biosynthesis of unsaturated fatty acids - Homo sapiens (human) | 23 | 7 (30.4%) | 2.21E-10 | KEGG |
| Fatty acid, triacylglycerol, and ketone body metabolism | 153 | 13 (8.6%) | 8.43E-11 | Reactome |
| Metabolism | 2035 | 42 (2.1%) | 4.08E-11 | Reactome |
